# Defining “Ethical Mathematical Practice” Through Engagement with Discipline-Adjacent Practice Standards and the Mathematical Community

**DOI:** 10.1007/s11948-024-00466-4

**Published:** 2024-04-30

**Authors:** Rochelle E. Tractenberg, Victor I. Piercey, Catherine A. Buell

**Affiliations:** 1 Departments of Neurology; Biostatistics, Bioinformatics & Biomathematics; & Rehabilitation Medicine, Georgetown University, Suite 207 Building D, 4000 Reservoir Road NW, Washington, DC 20057 USA; 2 Department of Mathematics, Ferris State University, 1201 S. State Street, Big Rapids, Michigan, 49307 USA; 3 Department of Mathematics, Fitchburg State University, 60 Pearl Street, Fitchburg, MA 01420-2697 USA

**Keywords:** Ethical mathematical practice, Ethical practice standards, Mathematics community, Mathematical practice, Ethics in mathematics

## Abstract

This project explored what constitutes “ethical practice of mathematics”. Thematic analysis of ethical practice standards from mathematics-adjacent disciplines (statistics and computing), were combined with two organizational codes of conduct and community input resulting in over 100 items. These analyses identified 29 of the 52 items in the 2018 American Statistical Association Ethical Guidelines for Statistical Practice, and 15 of the 24 additional (unique) items from the 2018 Association of Computing Machinery Code of Ethics for inclusion. Three of the 29 items synthesized from the 2019 American Mathematical Society Code of Ethics, and zero of the Mathematical Association of America Code of Ethics, were identified as reflective of “ethical mathematical practice” beyond items already identified from the other two codes. The community contributed six unique items. Item stems were standardized to, “The ethical mathematics practitioner…”. Invitations to complete the 30-min online survey were shared nationally (US) via Mathematics organization listservs and other widespread emails and announcements. We received 142 individual responses to the national survey, 75% of whom endorsed 41/52 items, with 90–100% endorsing 20/52 items on the survey. Items from different sources were endorsed at both high and low rates. A final thematic analysis yielded 44 items, grouped into “General” (12 items), “Profession” (10 items) and “Scholarship” (11 items). Moreover, for the practitioner in a leader/mentor/supervisor/instructor role, there are an additional 11 items (4 General/7 Professional). These results suggest that the community perceives a much wider range of behaviors by mathematicians to be subject to ethical practice standards than had been previously included in professional organization codes. The results provide evidence against the argument that mathematics practitioners engaged in “pure” or “theoretical” work have minimal, small, or no ethical obligations.

## Introduction

On the one hand, a mathematician is somebody who solves a problem or proves a theorem and, of course, publishes it. And it's hard to see significant ethical content in improving the value of a constant in some formula or calculating something new--say, the cohomology of some group... On the other hand, if you step back from that particular way of looking at the role of mathematicians and just think about your own activity or mine, think of what we actually do daily and yearly, there are constant decisions and conflicts involving right and wrong… The ethical demands of all the scientific groups seem to fall into three categories: What you owe the client, what you owe your profession, and what you owe the public. (Hersh, 1990, pp. 12–13)

Current (2024) discussions around “ethics in mathematics”, commonly include recent scandals and ethical vs. unethical algorithms (see, e.g., O'Neil, 2016). This is not to suggest that discussions on this topic are limited to these noteworthy examples, only to point out that popular discourse is widening the circle of mathematicians who are contemplating what “ethical practice” looks like, but other discussions about ethical mathematics are less scandal-driven (e.g., Müller, 2022 for recent discussion of “ethical mathematics” as “human activity” warranting ethical consideration; Karaali, 2019 for discussions of ethical obligations in the practice; and Dubbs, 2020, discussing ethical mathematics education research). In 1990, Hersh noted the efforts by physicists, chemists, biologists, and statisticians to develop codes of ethical practice, recognizing that their efforts reflect attention to ethical considerations “intrinsic to the actual practice of the particular profession” (p. 20). The stakeholders contemplated by Hersh in 1990 are important to the development of guidelines for ethical mathematical practice, because they are not delimited by quantitative *research* (e.g., Panter & Sterba, 2011), nor are they defined by their relationships to *applications* of mathematical practice. Hersh considered the profession–i.e., other practitioners—as a stakeholder in ethical mathematics. He closed his comments with the observation, “If our *research work* is almost devoid of ethical content, then it becomes all the more essential to heed our general ethical obligation as citizens, teachers, and colleagues, lest the temptation of the ivory tower rob us of our human nature.” (p. 23, emphasis added). We note that Hersh's (1990) assumption is that mathematical *research work* is “almost devoid of ethical content” (although see Müller, 2022; Ernest, 2021), and not that mathematical practice, nor the profession, is. This perspective is not echoed in consideration of the training of engineers to practice ethically: “the entire community of scientists and engineers benefits from diverse, ongoing options to engage in conversations about the ethical dimensions of *research* and (practice),” (emphasis added; Kalichman, 2013, p. 13).

Rittberg et al. (2020) focused on “the human role” in mathematical practice, relating to creativity and “the ways in which mathematicians perform their craft” (p. 3875). Specifically, they consider teaching and how injustice arises within the academic context in terms of access, validity, norms, and values of mathematics for both the instructors (authority/source of knowledge) and learners (receivers of knowledge). Ernest (2018) also discussed the ethical obligations of how we teach mathematics specifically. Educators have long been part of the conversation about *ethically teaching* mathematics (see e.g., Sowder, 1998; Neyland, 2004; Neyland, 2008; Atweh et al., 2012; Chiodo & Vyas, 2018; Piercey 2019). Part of the discussion about teaching ethical mathematics, or ethically teaching mathematics, has focused on the philosophy of mathematical practice, which “aims to understand mathematics, and potentially engage with how mathematics should be done” (Hamami & Morris, 2020; see also Dubb, 2020; Müller, 2022). Arguably, teaching mathematics is one type of mathematical practice. Our stance is that all of mathematical practice, and whether this comprises 100% or 5% of day to day work, can be done ethically if there is some sort of ethical guidelines specifically supportive of ethical mathematical practice. None of the literature on ethics in mathematics education (e.g., Müller, 2018, 2022; Stemhagen & Henney, 2021; Dubb, 2020; Chiodo & Clifton, 2019; Chiodo & Müller, 2018, Gustein, 2006) has featured formal evaluations of the impacts of these instructional initiatives however, and these efforts have not directly addressed the integration of authentic discussions of ethical content into our societies, research, and profession.

Bass (2006) articulated that engagement, particularly with research/scholarship in mathematics, comprises both a professional and a disciplinary aspect (pp. 103–104). A focus on integrating the concept of ethical mathematical practice into the undergraduate classroom represents a grass-roots approach to getting ethical practice into our societies and profession; if these efforts feed forward to graduate education, the chances increase of greater penetration into the profession and discipline. However, most of the literature on ethics and mathematics has been theoretical, or has presented discussion about why ethical issues are not addressed in typical mathematics courses or discourse, or why it is important to recognize ethical responsibilities in the practice of mathematics (although see Karst & Slegers, 2019; Miller, 2022). This project was designed to be empirical, and to generate tools as well as momentum for moving the conversation and the field forward.

Practice contexts, research/work, education, and engagement with the profession are fundamental elements of mathematical practice—i.e., “intrinsic to the actual practice of the particular profession” (Hersh, 1990, p. 20), and each comprise ethical considerations irrespective of the practitioner's role, area of mathematics, type of research, or career stage. These elements are also reflected in the construct of disciplinary stewardship (Ferrini-Mundy, 2008; Golde & Walker, 2006) and its cultivation (Rios et al., 2019). Henson et al. (2010) discussed a “collective quantitative proficiency” (CQP) model that explicitly prioritizes the authentic valuation of quantitative methods within the culture of a discipline that uses quantitative methodology. The CQP was described originally for education researchers, to encourage those who train doctoral students in education to more explicitly and consistently teach and apply quantitative methods.

The CQP construct was described as “a social consciousness that advances *quantitative concepts* as a logical extension of scientific inquiry and places value in training and orientation on the *interpretation of modern quantitative methods*” (p. 233; emphasis added). The argument and model can be seen to be appropriate to all sciences (Tractenberg, 2017). Chiodo and Bursill-Hall (2018) discuss a need for an “ethical consciousness” among mathematicians. Combining the Henson et al. *collective proficiency* concept–which brings with it an implied structure for teaching and assessing the target knowledge–with the “ethical consciousness” identified by Chiodo and Bursill-Hall, we suggest a *collective ****ethical**** proficiency* that can help to encourage and focus the growing and concerned interest in the ethical practice of mathematics.

Adapting the logic and construct from Henson et al., a *collective ethical proficiency* could benefit practitioners and learners by engaging practitioners and instructors in the inculcation of newcomers and trainees, leading to a new generation of ethically-knowledgeable practitioners. This situates “ethical mathematical practice” everywhere mathematical practice is perceived by practitioners to be relevant. What is needed is a definition of “ethical mathematical practice” around which the collective ethical proficiency can plausibly and consistently be formed.

Rather than concluding that mathematics has no content that could be subject to ethical practice standards beyond ethical scholarship and disciplinary preparation (AMS 2019), or beyond avoiding/managing conflict of interest (MAA, 2017), this study sought to explore the perceptions by the mathematical community of the ethical practice standards maintained by computing by the Association of Computing Machinery (ACM, since 1992) and the American Statistical Association (ASA, since 1995). We define “the mathematical community” to include anyone who identifies themselves as a member, rather than through any formal identification logic (e.g., outlined by Buckmire et al., 2023). In moving toward a collective ethical proficiency, rather than starting from scratch, mathematicians might leverage ethical practice guidelines from two fields intimately–and already—involved in the ethical use of quantitation and data: statistics and computing. Although they rely on foundational mathematics, each of these disciplines has aspects that are unique (see, e.g., Tractenberg, 2020). We thus also sought community input on additional ethical considerations apart from what computing and statistics practitioners and users have articulated, to ensure a description of “ethical mathematical practice” that is authentic as well as comprehensive.

## Review of Existing Codes

Scholars have debated the efficacy of ethics codes (see, e.g., Beauchamp & Bowie, 1979, Hoffman et al., 1984; Weller, 1988; see also McNamara et al., 2018), but before determining that codes do not work to promote ethical practice (e.g., McNamara et al., 2018; see also May & Luth, 2013; Antes et al., 2010), more and focused efforts are needed to teach and give practice with the use and utility of those codes (Tractenberg et al., 2015; Tractenberg, 2022b). A code–or set of ethical practice standards—articulates duties and responsibilities of a member of the profession that go beyond compliance with the law (Weller, 1988). Gillikin et al. (2017) define a “practice standard” as a document to “define the way the profession’s body of knowledge is ethically translated into day-to-day activities” (Gillikin et al., 2017, p. 1). Many mathematics practitioners might have variable engagement with mathematical practices in any given day; we do not include “as part of their daily work” (Buckmire et al., 2023) in our consideration of the applicability or relevance of ethical mathematical practice. Instead, this project cast a broad net for the varieties of work in which a mathematical practitioner might engage, and sought to offer ethical guidance for those using mathematical practice but not identifying as “mathematicians”, as well as for those who do identify as such. A code for ethical mathematics practice could be used to initiate and support the development of collective ethical proficiency among established practitioners as well as those in training, and for those who will use mathematical practices but never identify as, or hold a job with the title of, “mathematician”.

There is a wide range of member-societies with the goal of advancing mathematical sciences (see the Conference Board of the Mathematical Sciences 19 member organizations, https://www.cbmsweb.org/member-societies/https://www.cbmsweb.org/member-societies/). We focused on the American Mathematical Society (AMS) and the Mathematical Association of America (MAA) as they are the organizations with the broadest reach in the United States and they have each adopted some form of ethical principles. The AMS serves primarily mathematicians engaged in research as well as business and industry, while the MAA serves mathematicians who engage in both teaching roles and the scholarship of teaching and learning in higher education. Both societies have statements of ethics largely surrounding issues of plagiarism and publication (AMS) and conduct at meetings (MAA). Their statements are “Codes of Conduct” (which concerns individual behaviors in scholarship and at meetings) rather than “Ethical Guidelines” (see Müller et al., 2022)). Neither represents an “ethical practice standard” as defined by Gillikin et al. (2017). Tables 1 and 2 detail the themes represented in these codes.

**Table 1:** Thematic analysis results for AMS Code of Ethics elements

**I. Mathematical research and its presentation**
• Do not plagiarize, correct attribution when appropriate is essential
• Be knowledgeable in your field
• Give appropriate credit
• Do not claim a result in advance of its having been achieved; publish full details of results without unreasonable delay after announcing results
• Use no language that suppresses or improperly detracts from the work of others
• Correct or withdraw work that is erroneous
• A claim of independence may not be based on ignorance of widely disseminated results
• Ensure appropriate authorship
**II. Social responsibility of Mathematicians**
• ‡Encourage and promote mathematical ability without bias and review programs to ensure consideration of a full range of students
• Avoid conflicts of interest and bias in reviewing, refereeing, or funding decisions
• ‡Respect referee anonymity
• Resist excessive secrecy, promote dissemination/publication
• ‡Disclose implications of work to employers and the public when work may affect public health, safety, or general welfare
• *Do not exploit workers with temporary employment at low pay/excessive work)
**III. Education and Granting of Degrees**
• *Granting a degree means certifying competence for work
• ‡PhD level work is ensured by the degree grantors to be high level and original
• ‡PhD is only awarded to those with sufficient knowledge outside the thesis area
• *Degree grantors must honestly inform degree earners about job market/ employment prospects
**IV. Publications**
• ‡Editors should be reasonably sure of the correctness of articles they accept
• ‡Editors should ensure timely and current reviews
• ‡Submissions for review are treated as privileged information
• ‡Editors must prioritize the first submitted version of a paper
• ‡Editors must inform authors if there is a delay in potential publication
• ‡Publication cannot be delayed for any reason except the authors’ interest/actions
• ‡Date of submission and revisions must be published with any article
• ‡Editors must be given/accept full responsibility for their journals, resist outside agency pressures and notify the public of such pressure
• ‡Editors and referees must respect the confidentiality of all submitted materials as appropriate
• ‡Mathematical publishers must respect the mathematical community and disseminate work accordingly
• ‡The American Mathematical Society will not play a role/endorse any research journal where any acceptance criterion conflicts with the principles of the AMS guidelines

Note: ‡ identifies items were excluded from the survey (n=16). The 13 other items were only included if there was no concrete version of that item in ASA or ACM item lists. * indicates an item that is unique to ACM and was retained

**Table 2:** (Thematic) elements of MAA (MAA, 2017)

**I. Code of Conduct**
• ‡The MAA is committed to adhering to ethical business and professional practices, and to following a policy of honesty and integrity, in the full range of MAA activities
• ‡All employees of the MAA and all members engaging in the business, operations, and activities of the MAA shall adhere to all federal, state, and local laws and regulations and conduct themselves in a proper ethical manner
• ‡The MAA requires Directors, Officers, Members, those compensated by the MAA and those donating their time, and all employees to observe high standards of business and personal ethics in the conduct of their duties and responsibilities
• ‡All employees and representatives of the MAA must practice honesty and integrity in fulfilling their responsibilities and comply with all applicable laws and regulations
**II. Whistleblower policy**
• ‡The MAA will not tolerate intimidation, coercion, or discrimination of any kind against employees or other individuals who file complaints or who testify, assist, or participate in any manner in an investigation or hearing
• ‡It is the responsibility of all Directors, Officers, members and employees to comply with the Code of Ethics and to report violations or suspected violations in accordance with this Whistleblower Policy
• ‡No Director, Officer, member, or employee who in good faith reports a violation of the Code of Ethics shall suffer harassment, retaliation or adverse employment consequences
**III. Welcoming environment**
• ‡The MAA encourages the free expression and exchange of ideas in an atmosphere of mutual respect and collegiality. No discriminatory, harassing, or threatening by any staff member to any other person engaged in MAA operations or activities

Note: ‡ identifies items that were excluded from the survey if they applied to only a small subset of mathematics practitioners, limited contexts, or, if there were more concrete versions on other lists.

In addition to reviewing the codes of the AMS and MAA, we explored guidance from allied disciplines, statistics in the American Statistical Association (ASA) Ethical Guidelines for Statistical Practice (ASA, 2018; updated 2022) and computing through the Association for Computing Machinery (ACM) Code of Ethics (ACM, 2018). This study was initiated to gather input from mathematics practitioners across career stage, practice setting and role in the mathematical community through focused surveys. Instead of defining “mathematician”, we chose instead to adopt the perspective used by both the ASA and ACM. The ASA states in the preamble to its *Ethical Guidelines for Statistical Practice* (ASA, 2022), “… Throughout these guidelines, the term “statistical practitioner” includes all those who engage in statistical practice, regardless of job title, profession, level, or field of degree. The guidelines are intended for individuals, but these principles are also relevant to organizations that engage in statistical practice.” Similarly, the ACM states in its *Code of Ethics and Professional Conduct* (ACM, 2018), “ …The Code is designed to inspire and guide the ethical conduct of all computing professionals, including current and aspiring practitioners, instructors, students, influencers, and anyone who uses computing technology in an impactful way.”

Community input was sought to address the following questions about “ethical mathematical practice”:

1. Which elements of the existing ethical codes of the AMS and MAA are perceived to be relevant to ethical practice by the mathematics community?

2. What ethical guidelines from math-adjacent professional societies (ACM, ASA) does the mathematics community believe are relevant?

3. What other guidelines are necessary that are unique to mathematics? What ethical guidance is lacking from the AMS, MAA, ACM, and ASA guidelines?

We did not delineate membership in “the mathematics community” specifically, but rather issued an invitation to participate in the project to any individual who engages in mathematical practice, without limiting participation based on job title, profession, level, or field of degree.

## Methods

This project was granted IRB exemptions from all three participating institutions (Ferris IRB #FY19-20–205, Fitchburg IRB #202,021–14, and Georgetown IRB ID #00002454). The complete project report can be found at https://arxiv.org/abs/2209.09311.

### Item Selection

The project’s engagement with the mathematics community began with the creation of a preliminary set of items to be considered as part of a “Proto-Ethical Mathematics Practice Guideline” document. We began with all 52 items from the 2018 ASA Guidelines and the 24 ACM (2018) items. A thematic analysis carried out by the authors of the AMS (2019) and MAA (2017) codes, which are narrative, yielded 29 items for AMS and seven in MAA. The *stems* of items differ: ACM Code of Ethics (2018) items have the stem, “A computing professional should…” while the ASA Ethical Guidelines (ASA 2018) items have the stem, “The ethical statistician…”. AMS and MAA content, being narrative, do not include stems.

Table 1 presents the thematic analysis of the AMS code of ethics (AMS, 2019; the Code was updated in 2022 after this project was under way).

The Ethical Guidelines of the AMS (2019) were reviewed, and 13 elements (of 29 items abstracted from the Guidelines document) were retained for further consideration. The 16 items that are identified with the double cross in Table 1 were omitted from the survey by the authors for one of two reasons. Most typically they were highly limited to very few mathematics practitioners (e.g., “Editors should ensure timely and current reviews” relates only the Editor of AMS titles), so that they would be unlikely to be found relevant to ethical mathematical practice by respondents.

Ten of the 13 AMS items we identified were already reflected in specific items on the ASA Ethical Guidelines for Statistical Practice (ASA, 2018). The AMS Code is narrative (so our elements are the result of our own thematic analysis), while the ASA Guidelines are already broken into elements–and were specifically crafted for inclusion in the Ethical Guidelines by a specific Working Group of the Committee on Professional Ethics. Therefore, we utilized the ASA version of any item that is also reflected in one of the 13 AMS themes; endorsement of any ASA elements in the survey that also reflect an AMS item would be interpreted as endorsement of those AMS items. There were three AMS items (non-exploitation of workers; honest information about job prospects; certification of quality of Ph.D) that were not reflected at all on the ASA Guidelines, so we used our thematic analysis results as these AMS-specific items on the survey (indicated with an asterisk in Table 1).

The MAA Code of Ethics text was carefully reviewed by the authors and resulting “items” (themes) were deemed not sufficiently specific to ethical practice of mathematics to include in the survey. Any elements that were aligned with AMS, ASA, or ACM guidelines were retained as the AMS, ASA, or ACM item instead of the MAA theme.

The thematic analyses of the 110 items across the four ethical code documents yielded a preliminary sample of 86 items to be alpha and beta tested. *Alpha testing* occurred at the virtual 2021 Joint Mathematics Meeting (JMM; https://jointmathematicsmeetings.org/meetings/national/jmm2021/2247_intro) where attendees spent 1.5 h in subgroups considering subsets of the 86 elements. Fifty people joined this virtual Town Hall meeting where we separated them into six groups. The Town Hall meeting was advertised throughout the JMM program, and the session was open to any registered attendee of the 2021 JMM virtual meeting. Each group was assigned between 13 and 16 items from each of these source Guideline documents. Groups went through their lists and indicated whether (yes/no) that item would be considered relevant for “ethical mathematical practice”. Some additional items were recommended by attendees who thought the ideas were missing from the lists they were given.

*Beta testing* was accomplished when the authors reduced the starting number of survey items from 86 and any items that JMM 2021 Town Hall meeting attendees identified as important, but not already in the list of 86, down to a set of all those items that could be framed with respect to mathematics practice (i.e., by changing “computing professional” to “mathematics practitioner”, or by changing specifically statistics or computing terminology to be more consistent with mathematics instead). A small subset of the JMM 2021 Town Hall attendees agreed to be contacted for input on the content of this version of the survey. These beta testers helped us to ensure that the questions that we translated from the real-time Town Hall format to the asynchronous survey, made sense. Beta testers did not respond to survey items, only reviewed them. Beta testers ensured the link to the survey worked, commented on clarifications to the instructions, and also identified typos and other irregularities the authors missed.

*Final Survey*: At the time of the survey (2021), the ASA was revising its 2018 ethical guidelines (Tractenberg et al., 2021). Given input from the JMM 2021 Town Hall meeting, if items were identified there that had already been formulated for inclusion in the ASA revisions, we utilized the wording from the new ASA items in the beta test. Otherwise, we utilized wording from the JMM Town Hall meeting.

We winnowed the beta list down to 52 total items (plus demographics) after the removal of duplicates, and the determination of which of the beta version items were **unlikely** (in the authors’ or beta-testers’ opinions) to be viewed as relevant to the ethical practice of mathematics. Examples of items we omitted include, “(the ethical statistician) Employs selection or sampling methods and analytic approaches appropriate and valid for the specific question to be addressed, so that results extend beyond the sample to a population relevant to the objectives with minimal error under reasonable assumptions.” (ASA Ethical Guideline (ASA, 2018) Principle A2). The final 52-item survey (comprising 28 ASA, 14 ACM, 4 Town Hall, 4 AMS, and 2 hybrid ASA/ACM; ASA/Town Hall items) was deployed, with organization permissions, by sharing the survey invitation and URL link to it on SurveyMonkey through online messaging boards for members of several professional organizations including the AMS, MAA (including the Business, Industry, and Government Special Interest Group of the MAA), the Society for Industrial and Applied Mathematics (SIAM), and the American Mathematical Association of Two Year Colleges (AMATYC). That is, anyone who received emails from these groups, or who saw online messages from any of these groups, would have seen the open invitation to participate in this survey as part of the “mathematics community”. In our directions for completing the survey, we defined “mathematical practice” very generally, *“(w)e define the practice of mathematics to include mathematical work; the context in which or for which the work is done; the role of the practitioner; and the matter to which the mathematical work is directed or applied.*” We did not ask respondents to describe how or why they considered themselves to be part of this community, treating their awareness of the survey and interest in contributing as sufficient evidence of engagement with mathematics practice and membership in the target community.

Prefacing the survey was the following statement:

*The items are derived from several sources, so there is a bit of redundancy, but generally speaking, the items can be grouped as reflecting diverse elements of ****mathematical practice****. We define the practice of mathematics to include mathematical work; the context in which or for which the work is done; the role of the practitioner; and the matter to which the mathematical work is directed or applied. The survey asks you to consider whether each of the following items is relevant to the practice of mathematics.*

*Answer YES if you feel the item is an ethical obligation for the ethical mathematics practitioner. Answer NO if you feel the item is relevant, but not an ethical obligation; OR, if you feel the item is not relevant to ethical mathematical practice. We have included an option for you to comment on your answer.*

*Be sure to consider yourself as a mathematics practitioner, but also other practitioners in the mathematical community who may have different roles than you.*

All items in the survey had the same stem, “The ethical mathematics practitioner:”, for example, the first item would be read as:

**“The ethical mathematics practitioner:**

1. Works in a manner intended to produce valid, interpretable, and reproducible results.”

To increase interpretability of the survey results, we formulated the survey questions to include just “yes” and “no” answers (rather than a Likert scale of respondent-perceived relevance for each item), asking individuals to simply state whether or not they believe each item (given the stem, “the ethical mathematics practitioner”) was or was not “relevant to the practice of mathematics.” Each item also included the opportunity to comment on either the item or the participant’s response. One final item, “Please describe what you think is missing from the preceding list of items”, was also included.

With the exception of demographics, responses of “Yes” represent agreement/endorsement of an item: i.e., “YES if you feel the item is an ethical obligation for the ethical mathematics practitioner.” We contemplated how best to present the endorsement rates by item, including a simple tabulation and grouping items by endorsement level in order to better understand community thoughts about the relevance of each item for a new set of guidelines for “ethical mathematical practice”. Ultimately we utilized simple cluster analysis (in R) based on the endorsement values, and also studied the comments and missingness patterns in the data. Since the formal analysis did not yield interpretable results, we determined the final grouping based on our shared understanding of “general consensus” to be represented by 85–100% endorsement. The lowest levels of agreement would comprise 0–69.9% endorsement, and thus our middle range of endorsement ended up being 70.0–84.9%.

### Item-Level Analysis

Summary of survey items was based on the numbers of respondents who endorsed each item. We also conducted informal thematic analyses of both the comments on each item and the single open-ended survey item, “Please describe what you think is missing from the preceding list of items”. Finally, once survey results were tabulated, we reviewed the list of 52 elements to a) discuss consolidation rather than elimination, where feasible or items we or respondents deemed redundant; and b) identify duplication or confusion within and across items as noted by respondents.

## Results

A White Paper describing the entire year-long project (Buell et al., 2022) gives all results in fuller detail.

### General and Demographic Results

The survey was open for responses for three months. A total of 142 individuals completed the survey. Their demographics are presented in Table 3 (subtables A-E) below.

**Table 3:** Subtables A-E describing respondent demographics (A: Workplace; B: Highest degree; C: Years of Experience; D: Gender; E: Ethnicity)

A. Workplace or student status	Frequency	%
Non-Academic, Business/Industry	9	6.3%
Non-Academic, Government	4	2.8
Academic, Two-Year College Post-Doc, Faculty, or Administration	16	11.3
Academic, Four-Year, Primarily Undergraduate College/University Post-Doc, Faculty, or Administration	54	38.0
Academic, Ph.D Granting Institution Post-Doc, Faculty, or Administration	26	18.3
Academic, K-12 Teacher	6	4.2
Graduate student	8	5.6
Undergraduate student	6	4.2
Missing	13	9.3

B. Highest degree	Frequency	%
Bachelors	4	2.8
Ed.D	1	0.7
Masters	9	6.3
Ph.D./Ph.D in progress	82	57.7
Other	3	0.7
Missing	43	30.3

C. Years of experience	Frequency	%
0—5	17	12.0
6—10	10	7.0
11—15	7	4.9
16—20	12	8.5
21—25	13	9.2
26—30	8	5.6
More than 30	31	21.8
Missing	44	31.0

D. Gender	Frequency	%
Man	57	40.1
Woman	33	23.2
Non-binary	3	2.1
Prefer not to disclose	5	3.5
Missing	44	31.0

E. Ethnicity	Frequency	%
Asian or Asian American	3	2.1
Black or African American	3	2.1
Hispanic or Latino	4	2.8
White or Caucasian	78	54.9
Prefer not to respond	5	3.5
Missing	49	34.5

### Item-Level Analysis: Endorsement

Of the 52 items, 50% (26/52) were endorsed by 85–100% of respondents, with 38% (20 items) being endorsed by 90–100% of respondents. A further 17 items (36.7%) were endorsed by 70–84.9% of the sample. Thus, 43/52 (82.7%) of the items on the survey were endorsed by at least 70% of respondents as being “an ethical obligation for the ethical mathematics practitioner”. All of the 13 items reflecting AMS content, plus four items suggested from the Town Hall, were perceived to be relevant to ethical practice by at least 70% of respondents from the mathematics community. Three items unique to the ethical practice standards of statistics (ASA), and four unique to ethical computing (ACM), plus two suggested by mathematics community members at the Town Hall, were endorsed by 38.8–69.9% of respondents. Table 4 presents the 52 items on the survey in the order in which they appeared, annotated according to their source document(s). Dark grey shading shows the 26 items with the higest level of endorsement (85–100%). Light grey shading shows the 17 items with middle level endorsement (70–84.9%), while no shading (white) reflects the nine items with lowest levels of endorsement (38.8- 69.9%).

**Table 4:**
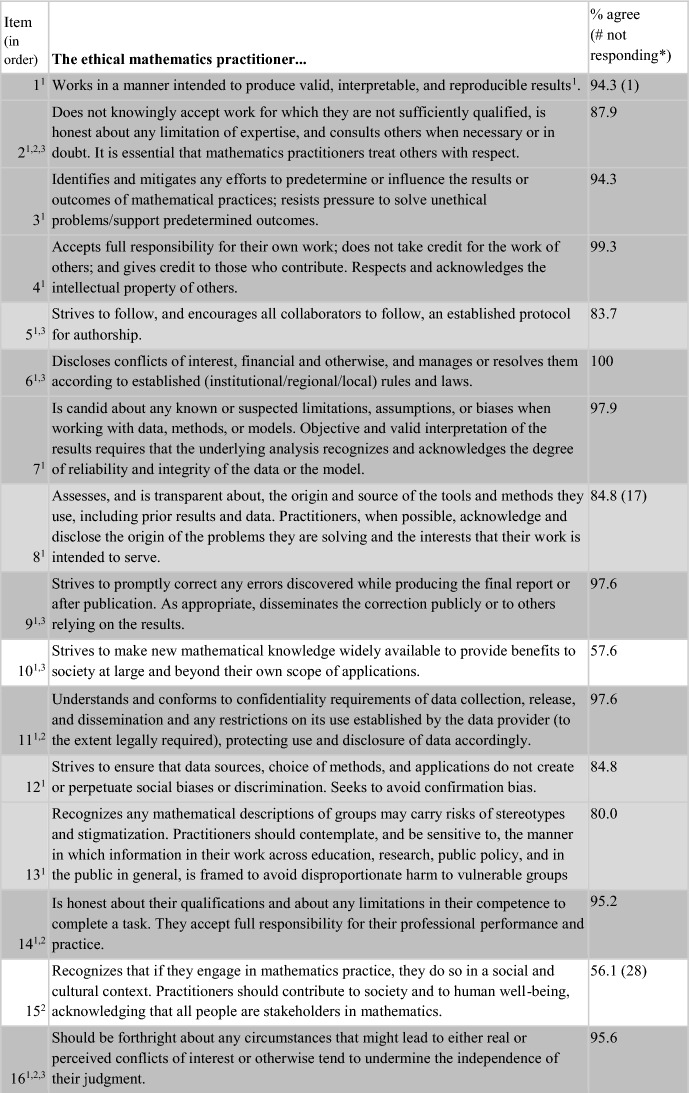

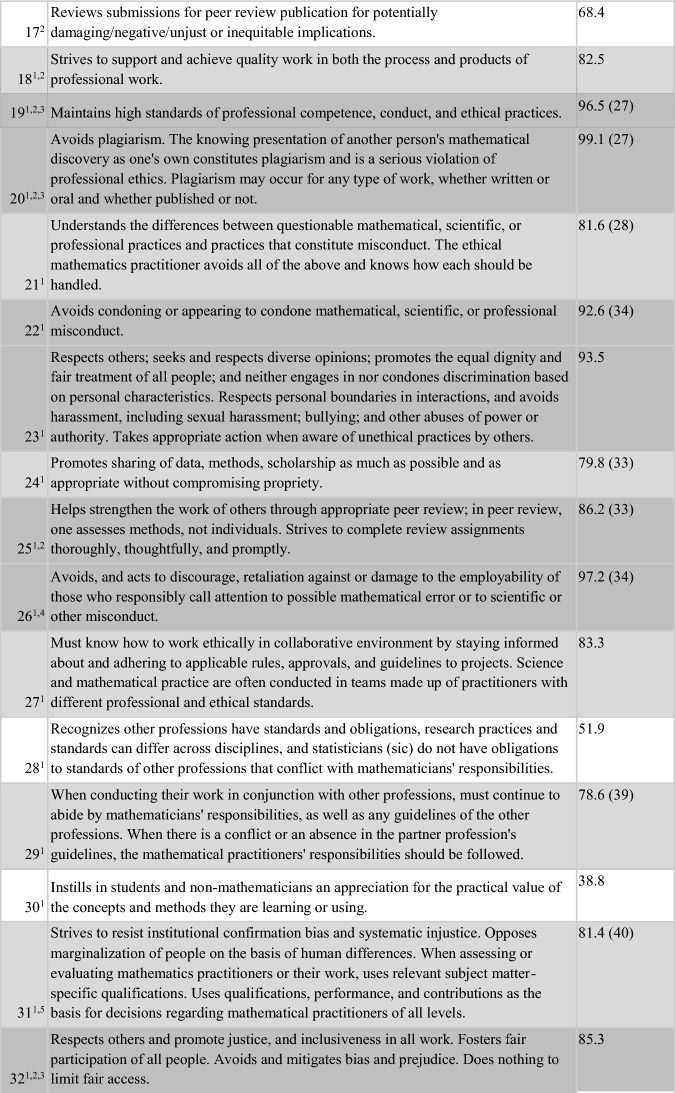

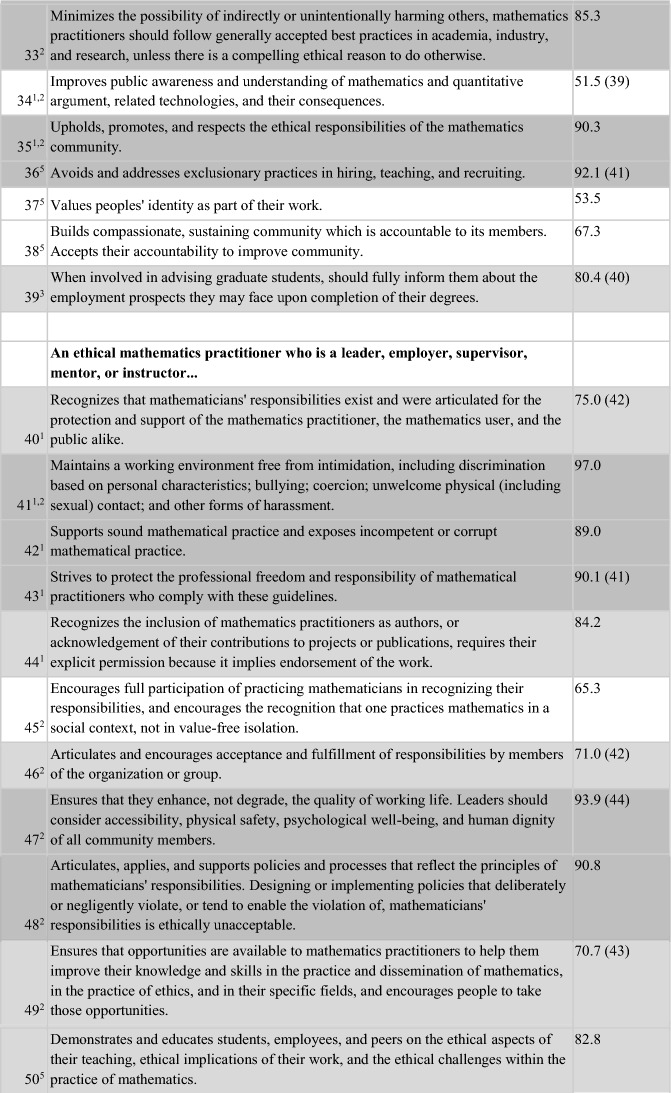

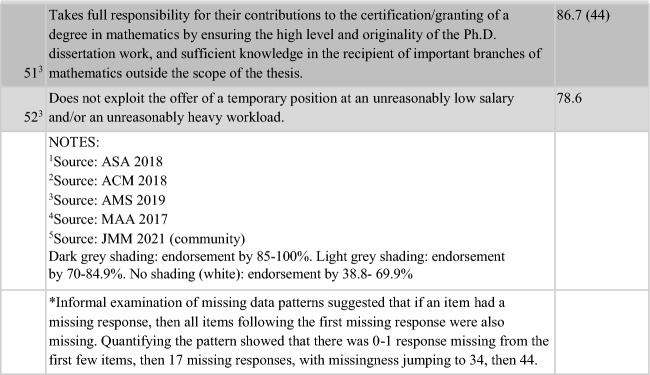
Results of the entire Ethical Guidelines survey, including the source(s) of each item

Of the 52 items, one had 100% endorsement: “Discloses conflicts of interest, financial and otherwise, and manages or resolves them according to established (institutional/regional/local) rules and laws.” This item wording came from the ASA Ethical Guidelines and was the only item with unanimous responses (although several others had endorsement at 99%, including “Accepts full responsibility for their own work; does not take credit for the work of others; and gives credit to those who contribute. Respects and acknowledges the intellectual property of others” and “Avoids plagiarism”), but the theme is also mentioned on the codes of the AMS, and ACM (albeit less explicitly).

### Respondent Suggestions for Additional Items

In response to the open-ended item, “Please describe what you think is missing from the preceding list of items”, we received 39 unique responses, several with > 1 item listed. Of these 39 responses, nearly half (18) did not include specific items that respondents thought were missing (examples of these comments were “nothing is missing” and “You treat the word “Ethical” as if it (is) rigidly, naturally or easily defined. It is not”). 11/39 respondents commented about clarity of items, or the perspective (did not suggest additional items). 8/39 respondents indicated additional elements were needed that were relevant to teaching/education specifically. 6/39 were not interpretable (e.g., “connection to related professional societies’ ethical standards”). 3/39 were specific to the workplace (irrespective of what the work is); 2/39 (which included many suggestions) related to academic work (not teaching), including scholarship. 2/39 related to updating the ethical guidelines. 1/39 referred to comments made on other items earlier in the survey, but did not suggest anything was missing from the survey.

Informal thematic analysis of these responses to the open-ended item led to the following six general categories that represent domains/aspects beyond what might be considered to be the more abstract or objective topics of some mathematical work:

- Workplace (not teaching, even if you do teach at work)–basic human respect/rights non-violations
- Educating (if this is your primary job/role or if you teach only as part of mentoring/collaborating): teaching effectively, grading objectively; doing your best to promote learning.
- Scholarship (writing, reviewing, and correcting errors, even if you made them)–respect for other’s work and others’ input to your work.
- Respect for the profession/stewardship (apart from scholarship, ethical & objective reviewing)
- Mathematics in the world: effectively preparing learners & users of math; not gatekeeping.
- Recognizing and effectively/respectfully treating stakeholders in work, teaching, scholarship, use of math, and the profession.

These themes, in addition to generally describing the types of items that respondents suggested were missing from the combined items adapted from ASA and ACM codes, and itemized from the AMS code, could also be used to organize a new set of guidelines for ethical practice of mathematics. After considering the comments and responses to the question, “what is missing?”, we concluded that if respondents felt the first/first few items were unclear, or not relevant, or presented another challenge to responding, then that respondent would stop answering the survey items.

### Analysis of Comments on Individual Item-Levels

The table below summarizes the themes uncovered from the item-level comments. Respondents were invited to comment on their answer to (or on the wording of) each item. We treated comment content as a “theme” if it was observed in two or more comments on at least two items. The first column includes the comment theme we uncovered, the second includes the items in which the comment was made with the endorsement of that item in parentheses (Table 5).

**Table 5:** Thematic analysis of comments on items, listed in order of frequency

Comment theme	Item # (percent “YES”)
Certain terms are too vague/unclear/loaded/subjective **Notes: Comment observed for items with a wide range of endorsement rates (and origins). Some readers want algorithmic guidelines, while ethical guidelines typically need room for interpretation in order to be comprehensive**	19 (96.5%)
	1 (94.3%)
	47 (93.9%)
	22 (92.6%)
	48 (90.8%)
	35 (90.3%)
	42 (89.0%)
	51 (86.7%)
	32 (85.3%)
	33 (85.3%)
	27 (83.3%)
	18 (82.5%)
	21 (81.6%)
	31 (81.4%)
	29 (78.6%)
	52 (78.6%)
	41 (75.0%)
	46 (71.0%)
	38 (67.3%)
	37 (53.5%)
	45 (65.0%)
	15 (56.1%)
	**22 items**
Item doesn’t rise to the level of an ethical obligation **Note: Comment observed for items with a wide range of endorsement rates (and origins)**	1 (94.3%)
	43 (91.0%)
	35 (90.3%)
	42 (89.0%)
	32 (85.3%)
	8 (84.8%)
	5 (83.7%)
	50 (82.8%)
	18 (82.5%)
	24 (79.8%)
	49 (70.7%)
	38 (67.3%)
	10 (57.6%)
	15 (56.1%)
	37 (53.5%)
	34 (51.5%)
	30 (38.8%)
	**17 items**
Item does not apply to pure mathematics **Note: Most of these items (7 out of 10) are about items with support over 80%**	7 (97.9%)
	11 (97.6%)
	16 (95.6%)
	3 (94.3%)
	8 (84.8%)
	12 (84.8%)
	5 (83.7%)
	17 (68.4%)
	10 (57.6%)
	37 (53.5%)
	**10 items**
Standard is too difficult to meet (includes comments about external obstacles such as proprietary work as well as comments about standards that are intrinsically too difficult to meet such as identifying your own biases) **Note: All but 1 of these pertains to items with 80% or higher support**	20 (99.1%)
	7 (97.9%)
	9 (97.6%)
	3 (94.3%)
	8 (84.8%)
	12 (84.8%)
	21 (81.6%)
	10 (57.6%)
	**8 items**
Item could harm those that should be protected (such as vulnerable or minoritized faculty) **Note: All of these pertain to items with 80% or higher support**	14 (95.2%)
	1 (94.3%)
	23 (93.5%)
	36 (92.1%)
	42 (89.0%)
	2 (87.9%)
	31 (81.4%)
	39 (80.4%)
	**8 items**
OTHER: •Item contains 2 or more distinct guidelines (which may or may not contradict one another)/ Item is tautological/leading/suggests its own answer /wording of item is poor •Item is about managerial work, not mathematical work •The truth is more important than the potential for harm •Pure mathematics is not itself unethical •Item is not unique to mathematics • Item does not apply to all mathematicians	(8 items 4/2/2)
	(4 items)
	(3 items)
	(3 items)
	(2 items)
	(2 items)

Finally, we reviewed the totality of the survey results and the 52 items that were included. Based on endorsement rates and comments, we eliminated three items that all had < 52% endorsement, and clarified another three items. Two other items that had lower endorsement rates were determined to be less clear, and possibly more redundant, than we hoped, so we combined and streamlined these into a single item. We also combined pairs of similar/similarly worded items, and moved similar/similarly worded segments of original items into items that had matching themes for clarity and elimination of redundancy. The result was a total of 44 items (Table 6). As a final thematic analysis, we loosely grouped items into three main categories, “General” < relating to all/all aspects of mathematical work > , “Profession” < relating to the profession specifically > , and “Scholarship” < very broadly defined > . We also determined that, like the 2018 ACM Code of Ethics and 2022 ASA Ethical Guidelines for Statistical Practice, there were specific items for the individual practitioner (12 General, 10 Profession, and 11 Scholarship items) and 11 other items specifically for the practitioner in a leader/mentor/supervisor/instructor role (4 General/7 Professional).

**Table 6:** Final 44-item Proto Ethical Guidelines for Mathematical Practice

**The ethical mathematics practitioner…**	
**IN GENERAL**	
1. Is honest about their qualification to complete work they accept; articulates any limitation of expertise, and consults others when necessary or in doubt. They accept full responsibility for their professional performance and practice	
2. Treats others with respect. Promotes the equal dignity and fair treatment of all people, and neither engages in nor condones discrimination based on personal characteristics. Respects personal boundaries in interactions, and avoids harassment, including sexual harassment; bullying; and other abuses of power or authority. Takes appropriate action when aware of disrespectful behaviors by others	
3. Accepts full responsibility for their own work; does not take credit for the work of others; and gives credit to those who contribute. Respects and acknowledges the intellectual property of others	
4. Should be forthright about any circumstances that might lead to either real or perceived conflicts of interest or otherwise tend to undermine the independence of their judgment. Discloses conflicts of interest, financial and otherwise, and manages or resolves them according to established (institutional/regional/local) rules and laws	
5. Recognizes any mathematical descriptions of groups may carry risks of stereotypes and stigmatization. Practitioners should contemplate, and be sensitive to, the manner in which information in their work across education, research, public policy, and in the public in general, is framed to avoid disproportionate harm to vulnerable groups	
6. Avoids condoning or appearing to condone mathematical, scientific, or professional misconduct. Takes appropriate action when aware of unethical conduct by others	
7. Avoids, and acts to discourage, retaliation against or damage to the employability of those who responsibly call attention to possible mathematical error or to scientific or other misconduct	
8. Is informed about applicable laws, policies, rules, and guidelines; follows these unless there is a compelling ethical reason to do otherwise	
9. Must know how to work ethically in collaborative environment. When conducting their work in conjunction with other professions, must continue to abide by mathematicians' responsibilities, as well as any guidelines of the other professions. When there is a conflict or an absence in the partner profession's guidelines, the mathematical practitioners' responsibilities should be followed	
10. Respects others, and promotes justice and inclusiveness, in all work. Fosters fair participation of all people. Avoids and mitigates bias and prejudice. Does nothing to limit fair access	
11. Opposes marginalization of people on the basis of human differences. Strives to resist institutional confirmation bias and systematic injustice	
12. Minimizes the possibility of harming others; whether directly or indirectly, intentionally or unintentionally	
**AS A MEMBER OF THE PROFESSION**	
13. Strives to make new mathematical knowledge as widely available as is feasible	
14. Maintains high standards of professional competence, conduct, and ethical practices	
15. Recognizes that if they engage in mathematics practice, they do so in a social and cultural context, acknowledging that all people are stakeholders in mathematics	
16. In reviews, considers the potential for unjust or inequitable implications of the proposal or work	
17. Understands the differences between questionable mathematical, scientific, or professional practices and practices that constitute misconduct. The ethical mathematics practitioner avoids all of the above and knows how each should be handled	
18. Avoids and addresses exclusionary practices in hiring, teaching, and recruitin. When assessing or evaluating mathematics practitioners or their work, uses relevant subject matter-specific qualifications. Uses qualifications, performance, and contributions as the basis for decisions regarding mathematical practitioners of all levels	
19. Upholds, promotes, and respects the ethical responsibilities of the mathematics community	
20. Accepts their accountability to build an inclusive mathematics community that values its members	
21. When involved in advising graduate students, should fully inform them about the employment prospects they may face upon completion of their degrees	
**IN THEIR SCHOLARSHIP**	
22. Strives to support and achieve quality work in both the process and products of professional work. Works in a manner intended to produce valid, interpretable, and when applicable, reproducible results	
23. Identifies and mitigates any efforts to predetermine or influence the results or outcomes of mathematical practices; resists pressure to solve unethical problems/support predetermined outcomes	
24. Assesses, and is transparent about, the origin and source of the tools and methods they use, including prior results and data. Practitioners, when possible, acknowledge and disclose the origin of the problems they are solving and the interests that their work is intended to serve	
25. Strives to follow, and encourages all collaborators to follow, an established protocol for authorship	
26. Is candid about any known or suspected limitations, assumptions, or biases when working with methods, models, or data. Objective and valid interpretation of the results requires that the underlying analysis recognizes and acknowledges the degree of reliability and integrity of the method, model, or data	
27. Assesses, and is transparent about, the origin and source of the tools and methods they use, including prior results and data. Practitioners, when possible, acknowledge and disclose the origin of the problems they are solving and the interests that their work is intended to serve	
28. Strives to promptly correct any errors discovered while producing the final report or after publication. As appropriate, disseminates the correction publicly or to others relying on the results	
29. Understands and conforms to confidentiality requirements of data collection, release, and dissemination and any restrictions on its use established by the data provider (to the extent legally required), protecting use and disclosure of data accordingly	
30. Strives to ensure that data sources, choice of methods, and applications do not create or perpetuate social biases or discrimination. Seeks to avoid confirmation bias	
31. Avoids plagiarism. The knowing presentation of another person's mathematical discovery as one's own constitutes plagiarism and is a serious violation of professional ethics. Plagiarism may occur for any type of work, whether written or oral and whether published or not	
32. Promotes sharing of data, methods, scholarship as much as possible and as appropriate without compromising propriety	
33. Recognizes the inclusion of mathematics practitioners as authors, or acknowledgement of their contributions to projects or publications, requires their explicit permission because it implies endorsement of the work	
**An ethical mathematics practitioner who is a leader, employer, supervisor, mentor, or instructor follows all of the above items and also…**	
**IN GENERAL**	
34.	Maintains a working environment free from intimidation, including discrimination based on personal characteristics; bullying; coercion; unwelcome physical (including sexual) contact; and other forms of harassment
35.	Articulates these ethical responsibilities to mathematics practitioners as well as non-practitioners
36.	Ensures that they enhance, not degrade, the quality of working life. Leaders should consider accessibility, physical safety, psychological well-being, and human dignity of all community members
37.	Does not exploit the offer of a temporary position at an unreasonably low salary and/or an unreasonably heavy workload
**AS A MEMBER OF THE PROFESSION**	
38.	Recognizes that mathematicians' ethical responsibilities exist and were articulated for the protection and support of the mathematics practitioner, the mathematics user, and the public alike
39.	Encourages and promotes sound and ethical mathematical practice, and exposes incompetent or corrupt mathematical practice
40.	Strives to protect the professional freedom and responsibility of mathematical practitioners who comply with these guidelines
41.	Articulates, applies, and supports policies and processes that reflect the principles of mathematicians' responsibilities. Designing or implementing policies that deliberately or negligently violate, or tend to enable the violation of, mathematicians' responsibilities is ethically unacceptable
42.	Ensures that opportunities are available to mathematics practitioners to help them improve their knowledge and skills in the practice and dissemination of mathematics, in ethical practice, and in their specific fields, and encourages people to take those opportunities
43.	Demonstrates and educates students, employees, and peers on the ethical aspects of their teaching, ethical implications of their work, and the ethical challenges within the practice of mathematics
44.	Takes full responsibility for their contributions to the certification/granting of a degree in mathematics by ensuring the high level and originality of the Ph.D. dissertation work, and sufficient knowledge in the recipient of important branches of mathematics outside the scope of the thesis

## Discussion

Our project was devised in order to answer three key questions to move efforts in the field forward with empirical, community-based, evidence. The survey yielded the following results:

1. Which elements of the existing ethical codes of the AMS and MAA are perceived to be relevant to ethical practice by the mathematics community?

Mathematics community members endorsed all of the 13 individual elements reflected on the AMS Code of Ethics that we included in our survey, 10 of which overlapped with items also included in the codes/guidelines of math-adjacent societies (ACM & ASA, which are the versions of the items that were used), yielding just three items unique to the AMS. As noted, our thematic analyses of the AMS and MAA guidelines led us to omit themes reflecting 100% of MAA and over 50% of AMS guidance. The primary rationale for these omissions was that the existing guideline elements were not relevant to “ethical mathematical practice” because they were too specific (e.g., to MAA employees or for highly delimited AMS editorial roles). One reason for this specificity of the MAA and AMS guidelines—which led to these omissions—might be that they reflect a larger community practice and belief that mathematical practice is inherently neutral and value-free; by this reasoning (if it was in play at all when the MAA and AMS codes were drafted), any ethical guideline would naturally have less to do with “mathematical practice” and more with specific roles (like MAA employment or AMS editorial duties). We saw some evidence of these two perspectives in comments on the survey, echoing some current scholarship (e.g., Ernest, 2021; Pearson, 2019; Shulman, 2002). The results strongly suggest community-level willingness to recognize ethical dimensions to mathematical practice.

p. 2. What ethical guidelines from math-adjacent professional societies (ACM, ASA) does the mathematics community believe are relevant?

We adapted 43 of the 52 items in the survey from the ACM and ASA ethical practice standards, 13 of which were also reflected on the AMS code. Thus, 32 items reflected aspects of ethical practice that are not included in current guidance from the AMS or MAA. Since we had to do a thematic analysis of MAA and AMS codes to create any items for the survey, we opted to use the more specific language of the ACM and ASA practice standards when AMS themes overlapped with those of the ASA or ACM. The process by which we created the survey suggests that future ethical practice standards for mathematics possibly should utilize a more elemental, less narrative, approach to guidelines. That is, in order to allow respondents to consider whether a given aspect of quantitative practice (survey item) represented an ethical obligation, it needed to be more elemental. In teaching “ethical mathematical practice”, particularly with case studies, elements (i.e., items) are likely to be more accessible for decision making than narrative text (see, e.g., Tractenberg, 2022b).

Importantly, every item on the survey was recognizable to respondents—to the extent that they were able to either endorse it as relevant to ethical mathematical practice, reject it, comment on it, or some combination of these. We interpret this to mean that a typical practitioner would be able to find specific guidance, and possibly justify a course of action, with a more elemental representation of their ethical obligations in any given case or aspect of practice. This may not be true for an oath or commitment to ethical practice. So, the results suggest that both specific content from, and the organization of, math-adjacent guidelines are relevant for the community.

Of the 52 items included, 51 of them were endorsed by more than 50% of the sample. The sole item that the majority did not endorse (only 38.8% endorsed it) was, “Instills in students and non-mathematicians an appreciation for the practical value of the concepts and methods they are learning or using.” This item was taken from the ASA Guidelines and modified—as all adapted items were-for mathematics; but we neglected to consider the role of “practical value” in the way mathematical concepts are viewed. Out of the 35 comments in the responses to this item, 11 specifically expressed objections to this idea. One commenter even compared mathematics work to arts and humanities (suggesting that no one would argue that the role of practical value in teaching the humanities should be an ethical obligation). While the idea of “practical value” has importance for statistics instruction, it has a different interpretation for mathematical practice and instruction. More generally, common reasons for the rejection of items by individual participants, based on our analysis of item-by-item comments, included the vagueness of terms, concern that an item was desirable but did not constitute “an ethical obligation”, and the perception that the item does not apply to “pure” mathematicians. This item was ultimately eliminated from our final version.

The ACM and ASA practice standards have been developed and refined by groups with the sole purpose of contemplating the wording, and applicability, of ethical guidelines to both the practitioner and the practice itself; so going forward, these results suggest that useful input from these ethical practice standards in terms of both content and the organization of elements (rather than narrative) can fruitfully be leveraged in the development of new guidelines for ethical mathematical practice. We asked respondents to consider both what *they* do, and the profession itself, in their consideration of whether an item is relevant to “ethical mathematical practice”. In our own discussions of the thematic analyses of comments, we determined that at least some of the reasons for those in the minority that did not endorse an item reflected a need for balance in the drafting of guidelines: precision vs. flexibility in terms, finding the right minimum standard. These are worthy of further discussion. Other reasons, such as “item does not apply to pure mathematics” or “the item is managerial and not mathematical” relate to considerations of professional identity and the role of ethical guidelines for a profession rather than an individual. Our final version of the proto-Guidelines includes language specifically about the individual and diverse roles and responsibilities they have (in general, to the profession, and in their scholarship; as well as in leadership roles). The Working Groups that worked on the ethical practice standards of both the ASA and ACM dedicated extensive time and effort to the wording, as well as item selection for inclusion. This effort will be required for new guidelines for ethical mathematical practice as well.

3. What other guidelines are necessary that are unique to mathematics? What ethical guidance is lacking from the AMS, MAA, ACM, and ASA guidelines?

Our analysis of the endorsement rates and item-level comments offered on each of the items suggested that there are important aspects of math-adjacent professions currently missing from existing guidance for ethical mathematical practice, but also highlighted important differences between disciplinary perspectives. For example, only one item had 100% agreement: “Discloses conflicts of interest, financial and otherwise, and manages or resolves them according to established (institutional/regional/local) rules and laws.” This was included in some form on all four source documents. Comments on 23 of the 52 items in the survey reflected a desire for greater precision of language, and potentially less opportunity for subjectivity in the articulation of ethical obligations. Comments on 17 of the 52 items suggested that the perception of an “ethical obligation” may differ slightly for mathematics as compared to statistics or computing. Comments on 10 of 52 items suggests that, for at least some respondents, there is a distinction between the ethical obligations incurred in “pure” mathematics and those incurred in other types of mathematics. These comments require discussion for the next iteration of these ‘proto guidelines’, but in no case did anyone suggest returning any item from AMS or MAA codes back into the proto-ethical guidelines.

Beyond the specific comments, in terms of “what was missing”, we noted six themes arising from the 39 suggestions for what was missing from our 52 items: workplace; teaching/grading/mentoring; scholarship; professional respect; effective preparation of users of mathematics (who are not mathematicians); and respect for stakeholders. The survey did not include any organization, but AMS, ASA, and ACM guidance documents all include subsections and organization. Our final version also includes more organization. The six themes arising from the analysis of these suggestions could be a useful addition to the organization of new guidelines for the ethical practice of mathematics. Moreover, the organization these themes suggest would also signal to practitioners that “ethical mathematical practice” is actually a complex set of behaviors that go well beyond “value-free” work–and apply to all practitioners in a variety of contexts. A critical limitation of efforts to develop codes to date is that their existence, and even an oath to commit to following any code (e.g., Müller et al., 2022), is that without specific practice following the code in authentic, work-related, circumstances, their function is highly delimited. Understanding how the knowledge behind the practice of mathematics is “ethically translated into day-to-day activities” is an essential aspect of buy-in from the wider community, but also how mathematics instruction could be modified to accommodate ethical content, and/or ethical reasoning, making the consideration of the impact of decisions in mathematical practice part of the “mathematical habits of mind” that instruction seeks to inculcate. We designed, and obtained US Federal funding, for a follow-on project which is focused on this specifically. In this project, undergraduate mathematics instructors from across the United States are actively creating content for their mathematics courses for all science, technology, engineering, and mathematics (STEM) fields, such as Calculus and Linear Algebra, to engage undergraduate mathematics students with content specific to ethical mathematical practice.

While we generated actionable answers to our research questions, important limitations to this study must be noted, chief of which is that we did not have a random sample of responses to our survey. Our respondents were all from institutions in the United States, but we do not know where they completed any of their training. Over half of the respondents have a PhD or are completing one, and one had an EdD; however educational achievement was missing from 40% of respondents. While we know that respondents had access to our invitations to participate (through the professional associations we were able to leverage), we cannot claim that this is a representative sample. Several aspects of the project counter this limitation; first among them is the high degree of concordance among our respondents for the majority of items on the survey. Moreover, the origins of the codes of ethics (MAA, AMS, ACM) and ethical guidelines (ASA) actually reflect much smaller cohorts (of 1–9 individuals). That is, the source documents we used to get our initial set of items arise from, and are maintained by, a small cohort of individuals charged specifically with the task of creating or revising/maintaining the ethical practice standard or code. While the ACM (2018) and ASA (2018/2022) explicitly sought input on the guidance documents from members of their respective organizations, it is not clear that the AMS or MAA have ever had constituent input on their codes. None of these organizations has ever conducted a survey like the one we created and deployed. Instead, these organizations selected a small cohort and tasked them with generating and/or revising their disciplinary ethical guidance. This survey is the first such assessment of community endorsement for any of these ethical practice standards. To our knowledge, it is the first empirical evidence about how individuals, for whom the invitation to participate in this survey resonated, perceive aspects of ethical mathematical practice. However, some of the comments we received in the Town Hall and on our survey have also appeared as anecdotes reported elsewhere (e.g., Chiodo & Clifton, 2019). This strengthens confidence in our conclusion that the survey results reflect community-level considerations.

Another limitation is that the ASA and ACM practice standards are comprised of specific elements, whereas the MAA and AMS codes are narrative. As noted, when there was overlap between MAA or AMS items with ACM or ASA items, we chose the ACM or ASA version to adapt for mathematical practice and the survey rather than utilize the results of our own, informal, thematic analysis of the AMS/MAA codes. It was easier to use/adapt the ASA and ACM items than the MAA and AMS codes, but we also had to adapt the majority of the ASA and ACM items and this was not always effective. Several commenters noted the poor wording or our accidental failure to omit “statisticians” in our adaptation of one item for mathematical practice. Our adaptations resulted in some awkward wording, apparent tautology, and other linguistic difficulties that were commented on by at least some respondents. These were addressed in our final content analyses leading to the set of 44 items.

A final limitation of this study is that there are no specific language relating to current specific challenges, such as AI, human rights, social justice, and sustainability within the ethical practice standard. Specific issues were not raised by any of our respondents in their comments, and because the ASA and ACM practice standards are meant to support all ethical practice (of statistics and data science, and computing, respectively), there are no mentions of specific issues in these source documents, either. However, several of the instructors in our current follow-on project have created new instructional materials that highlight specific issues or questions (e.g., social justice; environmental sustainability) in order to engage students in their mathematics courses in contemplating the impact of their work on others/these issues.

## Conclusions

This study described the first survey of mathematics practitioners in the United States to inquire about perceived relevance of elements of the AMS ethical code together with elements taken from math-adjacent professional societies for computing (ACM, 2018) and statistics (ASA, 2018).

Although some comments suggested that abstract aspects of mathematics may be incompatible with the applicability or utility of ethical practice standards or any of the items we included in our survey, Hersh’s three stakeholders and disciplinary stewardship should be considered by all practitioners in all contexts. We underscore our inclusive focus on “mathematics practitioners”, emphasizing that, like the ASA and ACM ethical practice standards, ethical guidelines for mathematical practice should not be limited in their applicability to solely those with the training or job title of “mathematician”. Instead, *anyone* who engages in, or contributes to, or utilizes the outputs of mathematical practices should be expected to do so ethically. Not all of the guidance elements will be relevant in every case, but a collective ethical proficiency could be based on at least a subset of the 51 items endorsed by the majority of respondents in this survey. The community's level of endorsement for a wide range of ethical obligations is empirical evidence against the argument that mathematics practitioners engaged in”pure” or “theoretical” work have minimal, small, or no ethical obligations (e.g., Hersh, 1990, p. 22; Müller, 2018; Müller et al., 2022, p. 40). The proto-guidelines described can be used to engage instructors in the inculcation of new members of the mathematics community, as well as those who study mathematics as part of their STEM disciplinary training.

Our survey was intended to answer specific questions about perceptions of relevance for ethical guidance from AMS and adjacent disciplinary standards, and can only be viewed as a first step in the effort in creating ethical practice standards for mathematics. Codes of conduct, and oaths (e.g., Müller et al., 2022) in particular are problematic because they treat the consideration of “ethical practice” as if it is static. Our choice of ASA and ACM source documents was purposeful, since these are plausible ethical practice standards for statistics and data science and for computing, respectively (Tractenberg, 2022b). But both resources also feature specific input for the individual and those in leadership roles. The 2022 ASA *Ethical Guidelines for Statistical Practice* also include an Appendix specific for those who employ statistical practitioners or utilize their work products. The ASA and ACM ethical practice standards are not static, and each reflects a disciplinary workflow that discourages consideration of “ethical practice” as a checklist or other fixed entity. These attributes of the long-standing ethical practice standards can be leveraged to introduce authentic engagement with “ethical practice of mathematics” for students, new practitioners, and leaders alike. A collective ethical proficiency can support a new generation of ethically-engaged practitioners, and this can be plausibly and consistently formed using the definition of “ethical mathematical practice” arising from the proto-guidelines that community respondents endorsed based on the guidelines from adjacent domains. Mathematics practitioners can leverage ethical practice standards that support statistics and computing in order to begin to formulate the basis–in terms of content, elemental organization, and thematic subsetting–for practice standards that promote a collective ethical proficiency in mathematics. Our team is currently exploring this with our cohort of 16 instructors of undergraduate mathematics courses. Some, but not all, of these projects are featuring the 44 proto Ethical Guideline elements. We have also proposed future workshops (2025) for stakeholders to utilize these proto-guidelines to create an action plan and a communication strategy for promoting a collective ethical proficiency among mathematics pracitioners in and from their specific contexts. The Guidelines are concrete and can be useful for creating cases for analysis, and for engaging students in discussions or reflections about “what does it look like to be an ethical mathematics practitioner” -which is useful for professional identity formation as well as for identifying employers with which students' ethical perspectives might align. They can be a tool to help mathematics practitioners participate in the vision where “the entire community of scientists and engineers benefits from diverse, ongoing options to engage in conversations about the ethical dimensions of *research* and (practice),” (emphasis added; Kalichman, 2013, p. 13).

## Data Availability

All data and survey materials are included in this manuscript.
